# U. S. Army Veterinary Notes

**Published:** 1890-08

**Authors:** 


### U. S. ARMY VETERINARY NOTES.

S. L. HUNTER, V.S., "Ontario" '87, is now employed as veterinary surgeon and professor of hippology at Fort Leavenworth, Kansas. He receives $125 a month, while other veterinary surgeons receive $75 to $100 a month.

Charles Douglas Mac Murdo, D.V.S., "A. V. C.," '89, has been appointed Jr. V.S., Seventh Cavalry, U. S. A., and is now stationed at Fort Sill, Indian Territory. J. A. W.

